# Protective effects of ginseng stem-leaf saponins on D-galactose-induced reproductive injury in male mice

**DOI:** 10.18632/aging.202709

**Published:** 2021-03-10

**Authors:** Qi Zhang, Chenying Yang, Min Zhang, Xiaomin Lu, Wanshuang Cao, Chunfeng Xie, Xiaoting Li, Jieshu Wu, Caiyun Zhong, Shanshan Geng

**Affiliations:** 1 Department of Nutrition and Food Safety, School of Public Health, Nanjing Medical University, Nanjing 211166, Jiangsu, China; 2 Center for Global Health, School of Public Health, Nanjing Medical University, Nanjing 211166, Jiangsu, China

**Keywords:** ginseng stem-leaf saponins, D-galactose, reproductive function, inflammatory response, oxidative stress

## Abstract

*Panax ginseng* is a perennial plant in the Araliaceae family. In this study, we investigated the protective effects of ginseng stem-leaf saponins (GSLS) isolated from *P. ginseng* against D-galactose-induced reproductive function decline, oxidative stress, and inflammatory response. Reproductive injuries were induced in mice via the subcutaneous injection of D-galactose (300 mg/kg) for six weeks. The mice were then treated with GSLS by intragastric administration. GSLS inhibited markers of oxidative stress and inflammatory cytokines induced by D-galactose in serum, liver and kidney, whereas GSLS increased the activities of antioxidant enzymes. Compared to the mice treated only with D-galactose, GSLS treatment significantly increased the average path velocity, straight line velocity, curvilinear velocity, and amplitude of the lateral head displacement of mouse sperm. Meanwhile, GSLS significantly increased the testosterone level and reduced the cortisol, FSH, and LH levels. Histopathological examination revealed alterations in the number and the arrangement of spermatogenic cells in the seminiferous tubules of the mice in the GSLS group. GSLS treatment suppressed MAPKs pathway activation in testes. These results suggest that GSLS can attenuate D-galactose-induced oxidative stress and inflammatory response in serum, liver and kidney, and ameliorate reproductive damage by inhibiting MAPKs signaling pathway.

## INTRODUCTION

Male reproductive function is known to decline with age. Aging accelerates genetic and epigenetic alterations in sperm, which can lead to diminished semen quality and reduced fertility [1]. Hassan et al. reported that after adjusting for the woman’s age, men over age 45 take five times longer to achieve pregnancy than men under age 25 [2]. Another large-population study found that compared to younger men, men over age 35 had a lower chance of impregnating their partners in 12 months of attempting conception after adjusting for maternal age [3].

The mechanism of reproductive dysfunction resulting from aging is not fully understood. Previous research has shown that increased oxidative stress may play a role in age-dependent reproductive dysfunction. Aging has been shown to result in reduced fertility, loss of Sertoli and germ cells in mice, excess spermatocyte reactive oxygen species (ROS), and DNA damage [4, 5]. Muratoğlu et al. reported that the content of malondialdehyde (MDA) in the testicular tissue was significantly higher in rats aged 20–22 months compared to in four-month-old rats [6]. The overexpression of the antioxidant enzyme catalase has been shown to have protective effects against age-related oxidative stress and decreased sperm production. Aged mice overexpressed with catalase (CAT) exhibited reduced ROS and peroxynitrite levels in their spermatocytes compared to the wild type, and the CAT mice did not exhibit the aging-associated loss of spermatozoa or testicular germ and Sertoli cells seen in the wild type [7]. Inflammation response has been proposed as a potentially important aging-related mechanism. Correlations have been found between inflammatory response and reduced sperm quality, including sperm count, motility, and morphology. Kahn et al. found that a systemic inflammatory response caused by obesity resulted in increased ROS and sperm DNA fragmentation [8]. Leisegang et al. recently found that the inflammatory cytokines TNF-α, IL-1β and IL-6 generated dose-dependent declines in steroidogenesis in Leydig cells [9].

The D-galactose (D-gal)-induced aging model has been widely used in aging research. It is proposed to be an agent that induces aging in animal because it can lead to a similar physiological state of aging, such as reduced antioxidant activity, increased oxidative stress and inflammation in testes, brain, liver, kidneys, and so on [10–13]. Liao et al reported that chronic administration of D-gal (6 to 8 weeks) can cause reproductive system damage, a decrease in superoxide dismutase activity and an increase in lipid peroxidation in testes, and a decline in testicular weight/body weight and sperm count [14]. Thus, D-gal is often used to induce aging in mouse model to evaluate the therapeutic effects of active compounds on inflammatory injury and oxidative stress.

*Panax ginseng* is a perennial plant in the Araliaceae family that is included in the Chinese Pharmacopoeia. The underground roots of *P. ginseng* are used as medicine after drying. In addition to the root, the stem-leaf of *P. ginseng* also contains ginseng saponins, which exhibit important pharmacological activities including antioxidant, anti-inflammation, and anti-aging effects. Zhang et al. analyzed the ginsenosides of different ginseng tissues using ultra-performance liquid chromatography–ultraviolet–mass spectrometry and found that the ginsenoside content decreased in the following order: leaf > rhizome > main root. Furthermore, the ratio between protopanaxatriols (Rg1, Re and RF) and protopanaxadiols (Rb1, Rb2, RC and Rd) in leaf increased from 1.37 to 3.14, whereas it remained stable at ~1.0 in the main root [15]. Recently, Lee et al. reported that ginseng stem-leaf saponins (GSLS) had highest amounts of Re [16]. These ginsenoside monomers, such as 20(R)-Rg3, F1, Rg1, Rb1, have been reported to have anti-aging effects, the mechanism of which is related to anti-inflammation, anti-oxidation and anti-apoptosis [17–21].

Previous investigations have demonstrated that GSLS might be a promising vaccine adjuvant against infectious disease [22, 23]. For example, upon the addition of GSLS, toxoplasma gondii heat shock protein 70 induced a strong immune response and provided partial protection against toxoplasmosis in mice [24]. Meanwhile, some studies have indicated that GSLS exhibits antioxidant activity. *In vitro*, ginseng leaf has been shown to have antioxidant capacity based on the 2,2-diphenyl-1-picrylhydrazyl, 2,2'-azino-bis(3-ethylbenzothiazoline-6-sulfonic acid), and hydroxyl radical scavenging activity methods [15]. Hu et al. found that ginsenosides from the stem-leaf of ginseng prevent ethanol-induced lipid accumulation related to the inhibition of oxidative stress in human LO2 hepatocytes [25]. Another study demonstrated that the oral administration of GSLS inhibited oxidative stress induced by cyclophosphamide by increasing the total antioxidant capacity, antioxidant concentration, and antioxidant enzyme activity while decreasing the levels of lipid peroxidation and the protein carbonyl content in chicken [26]. However, few works have attempted to clarify the protective effects of GSLS against reproductive injury caused by D-galactose (D-gal), oxidative stress, and inflammation. The purpose of this study was to examine the possible effects of GSLS on reproductive toxicity induced by D-gal and their underlying mechanisms.

## RESULTS

### Effects of GSLS on body weight and liver and kidney function

**Figure 1:**
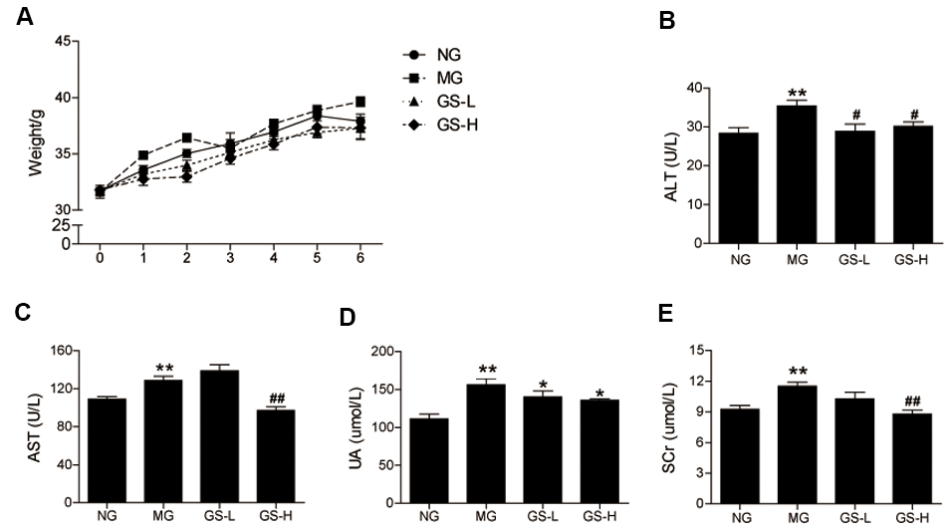
**Effects of GSLS on body weight and liver and kidney function.** (**A**) body weight; (**B**) alanine-aminotransferase (ALT); (**C**) aspartate-aminotransferase (AST); and (**D**) uric acid (UA); (**E**) serum creatinine (Scr). All data are represented as mean ± SEM (*n* = 6). *p < 0.05, **p < 0.01 compared to the NG; ^#^*p* < 0.05, ^##^*p* < 0.05 compared to the MG. NG: normal group, MG: D-gal model group, GS-L: D-gal + low dosage of GSLS group, GS-H: D-gal + high dosage of GSLS group.

As shown in Figure 1A, no significant differences in body weight were observed between the four groups during the six-week intervention. The effects of GSLS on liver and kidney function in D-gal-induced aging mice were examined based on the measured levels of ALT, AST, UA, and SCr in serum. As shown in Figure 1B–1E, the MG mice exhibited a marked increase in the levels of ALT, AST, UA, and SCr (*p* < 0.01) compared to NG mice. The levels of ALT, AST, UA, and SCr were significantly lower in the GS-L and GS-H groups than in the MG. These results demonstrate that GSLS are safe. GSLS do not effect body weight or burden the livers and kidneys of aging mice; they may even improve liver and kidney function.

### Effects of GSLS on oxidative stress markers and pro-inflammatory cytokines in the liver and kidney of D-gal-induced mice

**Figure 2:**
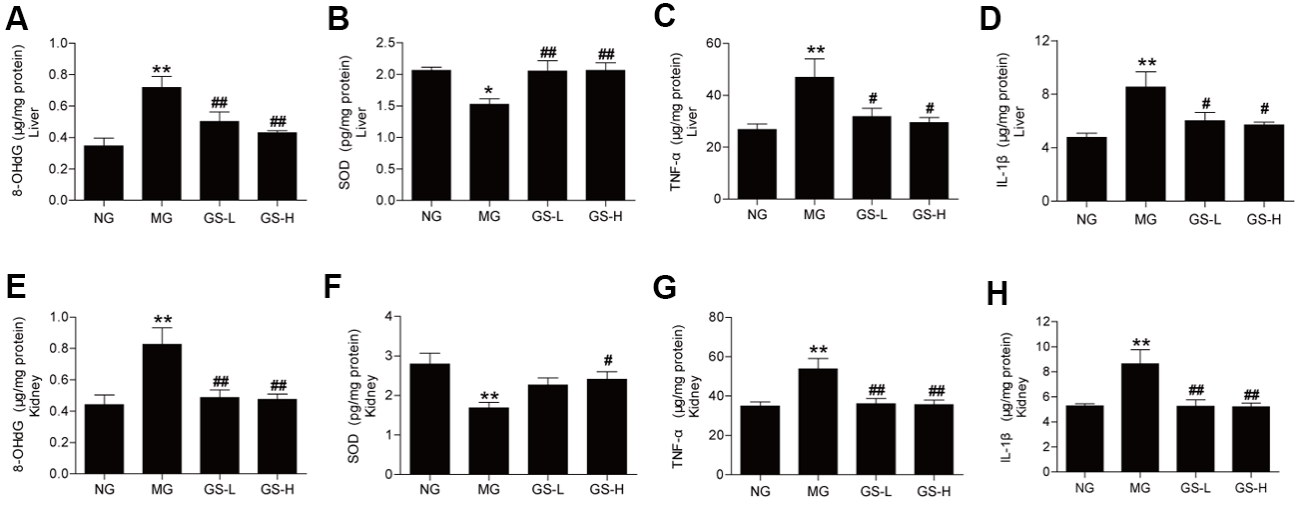
**Effect of GSLS on D-gal-induced changes in oxidative stress markers and pro-inflammatory cytokines in liver and kidney tissues.** (**A**) 8-hydroxy deoxyguanosine (8-OHdG) in liver; (**B**) superoxide dismutase (SOD) in liver; (**C**) tumor necrosis factor-α (TNF-α) in liver; (**D**) interleukin-1β (IL-1β) in liver; (**E**) 8-hydroxy deoxyguanosine (8-OHdG) in kidney; (**F**) superoxide dismutase (SOD) in kidney; (**G**) tumor necrosis factor-α (TNF-α) in kidney; (**H**) interleukin-1β (IL-1β) in kidney. All data are represented as mean ± SEM (*n* = 6). *p < 0.05, **p < 0.01 compared to the NG; ^#^*p* < 0.05, ^##^*p*<0.05 compared to the MG. NG: normal group, MG: D-gal model group, GS-L: D-gal + low dosage of GSLS group, GS-H: D-gal + high dosage of GSLS group.

To determine whether GSLS can attenuate the increased oxidative damages and inflammation in the liver and kidney tissues of D-gal-treated mice, we analyzed the 8-OHdG, SOD activities, IL-1β and TNF-α levels. As shown in Figure 2, GSLS could decrease the 8-OHdG levels and renew the activities of SOD both in the liver and kidney of D-gal-treated mice. And GSLS alleviated the levels of IL-1β and TNF-α in D-gal-treated liver and kidney tissues. These results illustrated that GSLS hindered the damage caused by D-gal-induced oxidative stress and inflammation in both the liver and kidney.

### Effects of GSLS on oxidative stress markers and pro-inflammatory cytokines in the serum of D-gal-induced mice

**Figure 3:**
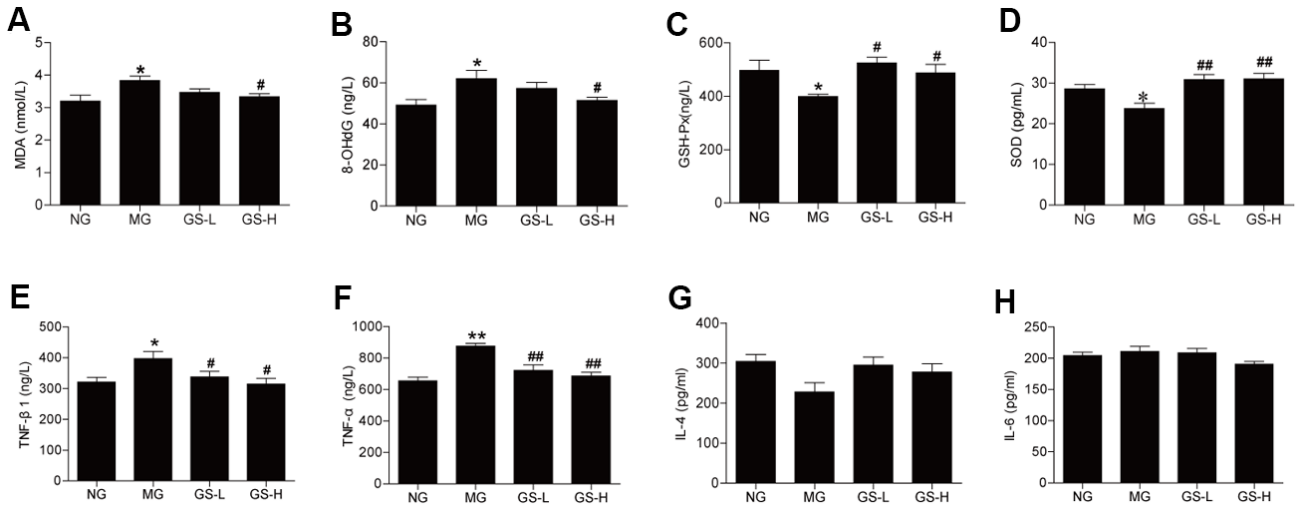
**Effects of GSLS on D-gal-induced changes in oxidative stress markers and pro-inflammatory cytokines in serum of D-gal-induced mice.** (**A**) malondialdehyde (MDA); (**B**) 8-hydroxy deoxyguanosine (8-OHdG); (**C**) glutathione peroxidase (GSH-Px); and (**D**) superoxide dismutase (SOD); (**E**) tumor necrosis factor-β1 (TNF-β1); (**F**) tumor necrosis factor-α (TNF-α); (**G**) interleukin-4 (IL-4); and (**H**) interleukin-6 (IL-6). All data are represented as mean ± SEM (*n* = 6). *p < 0.05, **p < 0.01 compared to the NG; ^#^*p* < 0.05, ^##^*p* <0.05 compared to the MG. NG: normal group, MG: D-gal model group, GS-L: D-gal + low dosage of GSLS group, GS-H: D-gal + high dosage of GSLS group.

The effects of GSLS on oxidative stress markers in mice serum were examined based on the measured levels of MDA, 8-OHdG, SOD and GSH-Px. The levels of MDA and 8-OHdG in serum were significantly elevated in MG mice compared to in NG mice (*p* < 0.05; Figure 3A, 3B). High-dose GSLS treatment significantly reduced the MDA and 8-OHdG levels compared to the MG. The levels of GSH-Px and SOD in serum were lower in MG mice than in NG mice (*p*< 0.05; Figure 3C, 3D). GSLS treatment significantly increased the levels of GSH-Px and SOD in serum compared to the MG.

As shown in Figure 3E, 3F, the concentrations of TNF-β1 and TNF-α were significantly higher in MG mice than in NG mice (*p* < 0.01), while D-gal did not have significant effects on the levels of IL-4 and IL-6 (Figure 3G, 3H). Treatment with GSLS significantly decreased the levels of TNF-β1 and TNF-α compared to the MG.

### Effects of GSLS on sperm motility in D-gal-induced mice

**Figure 4:**
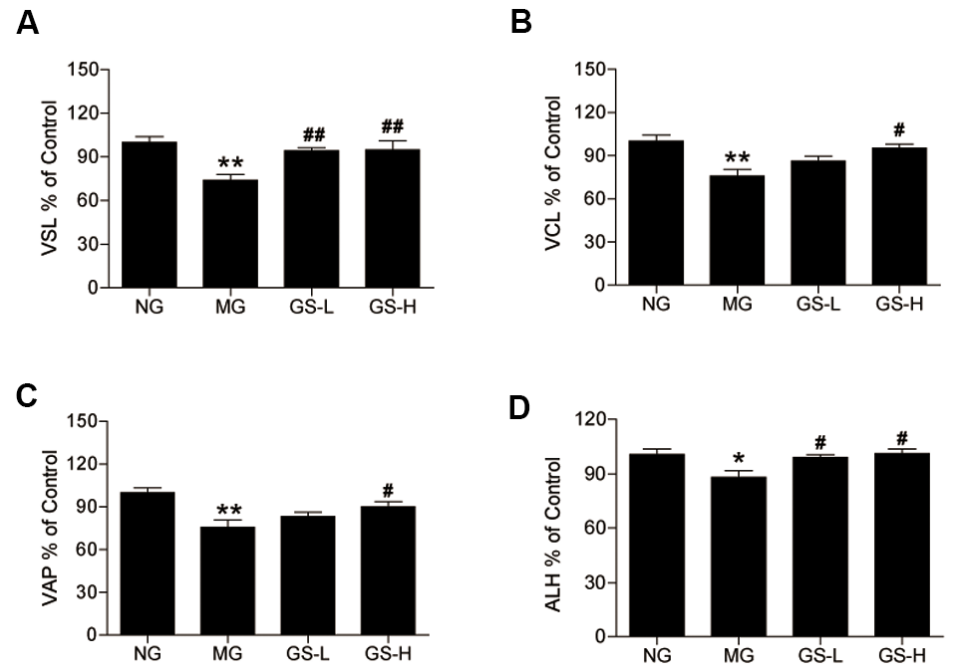
**Effects of GSLS on the kinematic parameters of mouse sperm.** (**A**) straight line velocity (VSL); (**B**) continuous line velocity (VCL), (**C**) average path velocity (VAP); and (**D**) amplitude of lateral head displacement (ALH). All data are represented as mean ± SEM (*n* = 6). *p < 0.05, **p < 0.01 compared to the NG; ^#^*p* < 0.05, ^##^*p* < 0.05 compared to the MG. NG: normal group, MG: D-gal model group, GS-L: D-gal + low dosage of GSLS group, GS-H: D-gal + high dosage of GSLS group.

As shown in Figure 4, all sperm motility parameters (VSL, VCL, VAP and ALH) were lower in MG mice than in NG mice. Treatment with GSLS significantly increased the levels of VSL, VCL, VAP and ALH in mouse sperm compared to the MG.

### Effects of GSLS on sex hormones in the serum of D-gal-induced mice

**Figure 5:**
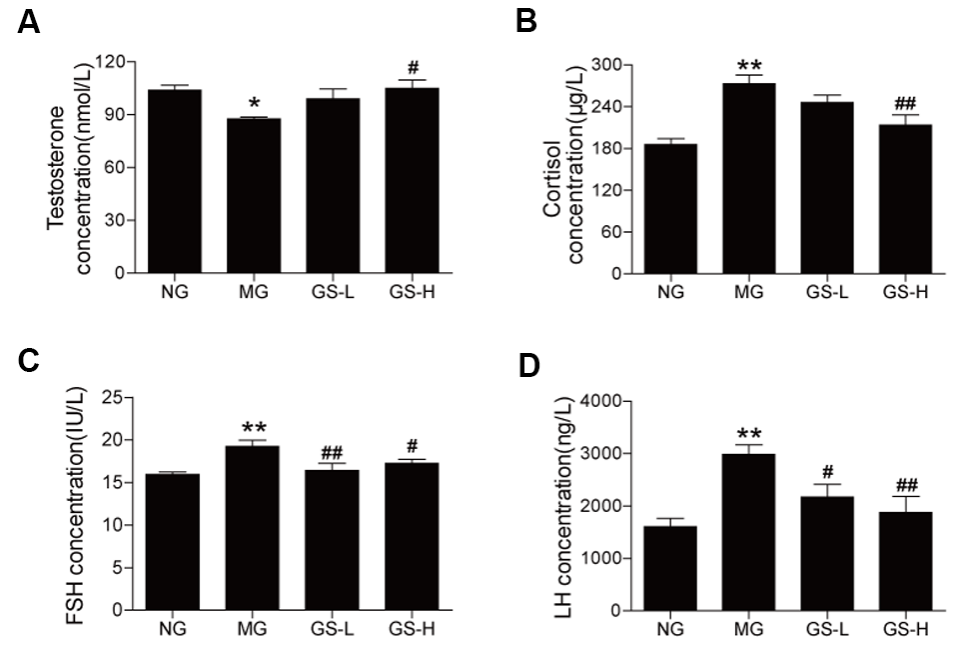
**Effects of GSLS on sex hormones in the serum of D-gal-induced mice.** (**A**) testosterone; (**B**) cortisol; (**C**) follicle-stimulating hormone (FSH); and (**D**) luteinizing hormone (LH). All data are represented as mean ± SEM (*n* = 6). *p < 0.05, **p < 0.01 compared to the NG; ^#^*p* < 0.05, ^##^*p* < 0.05 compared to the MG. NG: normal group, MG: D-gal model group, GS-L: D-gal + low dosage of GSLS group, GS-H: D-gal + high dosage of GSLS group.

The MG mice had a lower level of testosterone in serum than the NG mice (*p* < 0.05, Figure 5A). GSLS treatment significantly increased the testosterone level in the serum of mice compared to the MG. The levels of hormones (cortisol, FSH and LH) in the serum were significantly higher in MG mice than in NG mice (*p*< 0.05; Figure 5B–5D). GSLS treatment significantly decreased the levels of cortisol, FSH and LH compared to the MG.

### Effects of GSLS on pathological changes in testes of D-gal-induced mice

**Figure 6:**
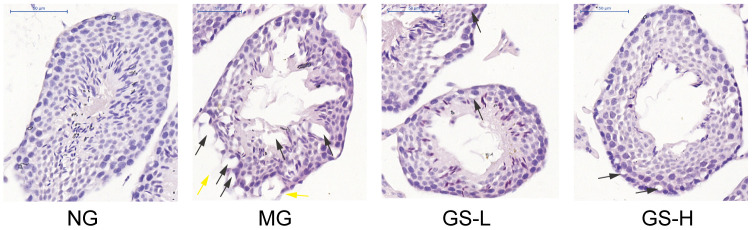
**Effects of GSLS on histological changes in testes of D-gal-induced mice.** Representative images at 400× magnification; bar indicates 50 μm. Black arrow head: Sertoli cell vacuolization. Yellow arrow head: interrupted basement membrane. n=3 per group. NG: normal group, MG: D-gal model group, GS-L: D-gal + low dosage of GSLS group, GS-H: D-gal + high dosage of GSLS group.

As shown in Figure 6, no histopathological change of seminiferous tubules was observed in the control group. Well-defined spermatogenic cells were lined on a thin basement membrane. The spermatogenic cells were arranged orderly and in clear layers. All levels of spermatogenic cells were observed. The Sertoli cells were normal in shape and embedded in the basement membrane and spermatogenic cells. In contrast, significant changes were seen in the testes of mice exposed to D-gal. The structure of seminiferous tubules was damaged, the vacuole was denatured, the spermatogenic epithelium became thinner and the number of layers decreased. The number of spermatogenic cells was decreased and the arrangement was loose. Compared with D-gal group, GSLS treatment improved the histological changes of testis and gradually increased germinal layer to restore spermatogenesis. While when compared with the control group, there was no mature sperm in the lumen. Taken together, D-gal treatment induced histopathological changes of testis, and GSLS intervention improved these alterations.

### Effects of GSLS on mitogen-activated protein kinase (MAPKs) pathway in testes of D-gal-induced mice

**Figure 7:**
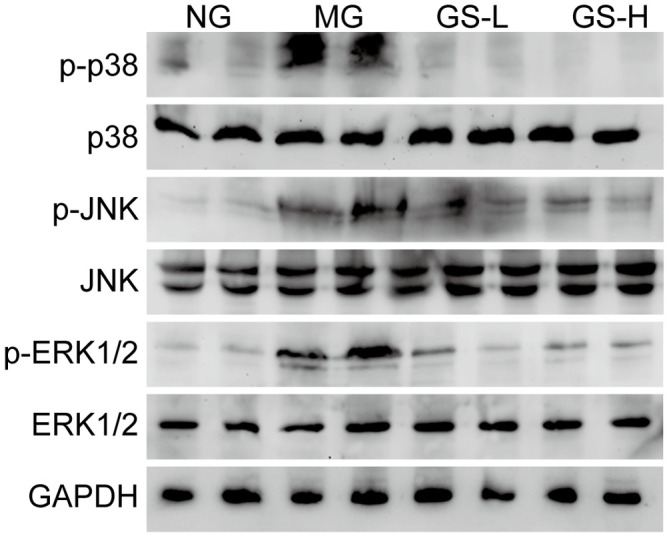
**Effects of GSLS on MAPKs signaling pathway in testes of D-gal-induced mice.** n=3 per group. NG: normal group, MG: D-gal model group, GS-L: D-gal + low dosage of GSLS group, GS-H: D-gal + high dosage of GSLS group.

The MAPKs pathway plays important role in regulation of gonadal hormone, sperm quality, morphological change [27]. To determine the protective effects of GSLS, we measured the expression of MAPK family members, including p-JNK, p-p38 and p-ERK, in the different groups. As shown in Figure 7, a greater increase in the phosphorylated forms of p38, JNK, and ERK1/2 was observed in MG mice than in NG mice. However, GSLS treatment significantly alleviated the D-gal-induced activation of MAPKs in the testes.

## DISCUSSION

For the first time, our results demonstrate the potential beneficial effects of GSLS on reproductive dysfunction induced by D-gal in mice. The results indicate that GSLS can protect the sperm, seminiferous tubules and sex hormones against damage, inhibit oxidative stress and inflammation to some extent.

Aging is known to be associated with damage to male reproductive function. Stone et al. analyzed the semen quality of 5,081 men aged 16.5–72.3 years and found negative correlations between age and various sperm parameters including semen number, semen concentration, normal sperm morphology rate, sperm motility, and progressive parameters of motile sperm [28]. Similarly, Verón et al. recently found age to be negatively correlated with routine semen parameters including sperm count, sperm volume, sperm motility, total motile sperm count, normal motile sperm count, round cell concentration, and hypo-osmotic swelling [29]. Although individual variation exists, the results provide evidence that semen parameters indicative of semen quality decline with age.

The most common animal models used to investigate male reproductive aging involve aging and D-gal- injected rat and mouse models [30–32]. D-gal is a reducing sugar usually found in the body. When the D-gal concentration exceeds normal level, it is converted to aldehydes and H_2_O_2_ [32, 33]. Animals injected with D-gal showed aging-related phenotypes, such as decreased immune responses, neurological defects, decreased antioxidant enzyme activity, increased ROS production and reproductive aging [14, 34–36]. Ahangarpour et al. used 500mg/kg D-galactose for six weeks and reported decreased sperm counts and increased serum LH levels in male mouse [31]. Liao et al reported that mice injected with 200mg/kg D-gal for 6 or 8 weeks demonstrated lower superoxide dismutase activity in the serum and testis lysates and increased testicular lipid peroxidation. In addition, testicular weight/body weight and sperm count were decreased in the D-gal group, and the percentage of abnormal sperm was increased significantly. C-DNA microarrays revealed abnormal gene expression associated with spermatogenesis in D-gal group. This study suggests that mice injected with D-gal are suitable for the study of male reproductive aging [14]. On the basis of the above studies, the present study also established a model of aging and reproductive injury in mice by D-gal injection. Our results revealed significant changes in sex hormones, sperm motility and testis histopathology in male mice. Epididymis is also one of the reproductive organs, which was collected in this study to measure sperm motility with Casa Instrument. Although the morphological changes in epididymis were not examined, the alterations in sperm motility and testis histopathology illustrated significant changes in D-gal injected mice.

The male hypothalamic–pituitary–gonadal axis controls the release of sex hormones and ensures the formation and maturation of spermatogenic cells. The axis consists of three essential components: the hypothalamus, anterior pituitary, and testis. In this axis, gonadotropin-releasing hormone (GnRH) secreted by the hypothalamus reaches the anterior pituitary gland via hypophyseal portal circulation and stimulates the secretion of LH and FSH in gonadotropic cells, which are then released into the bloodstream. LH induces the production of testosterone in Leydig cells, while FSH induces the production of androgen-binding protein and inhibin in Sertoli cells; thus, LH and FSH play an important role in initiation and progression of spermatogenesis. Extensive studies have shown that the levels of many hormones vary with age. Serum testosterone levels are known to decrease with age, accompanied by disruption of the diurnal rhythm of GnRH, decreased chorionic gonadotropin secretion, deteriorated testicular perfusion, and a decrease in Leydig cells. The pituitary gonadotropins FSH and LH were found to increase with age by 1.9% and 1.3% per year, respectively [37–39]. FSH was found to be negatively correlated with semen parameters (*p* < 0.05). Similarly, LH was found to be negatively correlated with total sperm count, normal sperm morphology, and abortion (*p* < 0.05). Our results showed that mice with D-gal-induced aging exhibited decreased sperm motility, viability, and serum testosterone along with increased FSH and LH levels after six weeks of treatment.

Hormones such as testosterone are often used as adjuvants, although various adverse reactions have been reported [40, 41]. Therefore, developing effective medicines is a core issue in treating age-related reproductive decline. Natural products have emerged as complementary and/or alternative medicines and show potential for therapeutic applications worldwide. The results of this study provide evidence that GSLS, which are flavonoids, protect aging male mice from reproductive dysfunction. In this study, D-gal-treated mice showed decreased sperm motility and viability along with increased FSH and LH levels after six weeks of treatment. The administration of GSLS resulted in increased sperm motility and testosterone levels compared to the D-gal-treated mice. This improvement may be related to free radical scavenging and reduced biochemical oxidative stress. Previous studies have also suggested that GSLS have antioxidant properties.

Oxidative stress, which results from an imbalance between the production of ROS and antioxidant scavengers, is an important process associated with age-related decline in male reproductive capacity. Growing evidence suggests that age-related oxidative stress is associated with male reproductive dysfunction resulting from decreased semen quality, endocrine changes, and decreased sexual function. Weir et al. reported that spermatozoa damage is related to increased levels of hydrogen peroxide and superoxide along with decreased activities of GSH-Px and SOD in aging rats [42]. Furthermore, aged SOD-null mice were found to have lower fertility and severe redox dysfunction compared to aged wild-type mice [5]. In contrast, the overexpression of the antioxidant catalase was found to alleviate age-related oxidative stress and sustain the spermatogenesis process in mice [7]. In the present study, GSLS treatment reduced the MDA level and protected the activities of SOD and GSH-Px in aging mice compared to D-gal-treated mice. Indeed, natural products containing GSLS have been reported to have protective effects against oxidative stress. Yu et al. showed that GSLS significantly inhibited oxidative stress induced by cyclophosphamide by increasing the total antioxidant capacity, the activities of total SOD, catalase, and GSH-Px, and reducing the contents of protein carbonyl and malondialdehyde in chicken [26]. Another study showed that GSLS alleviated hepatocyte steatosis induced by ethanol *in vitro* by reducing oxidative stress [25]. Therefore, the improvement in sperm parameters and sex hormones after treatment with GSLS in this study may be related to the suppression of oxidative stress based on the antioxidant properties of GSLS.

Inflammation, which can be caused by oxidative stress, is considered as a common predisposing factor in male reproductive decline [43]. TNF-α is a pro-inflammatory factor in both local and systemic inflammation. TNF-α plays a central role in the production of many inflammatory agents, including NO and ROS. TNF-α can directly or indirectly reduce spermatogenesis, destroy sperm membranes, and reduce semen quality. Pascarelli et al. reported that TNF-α had negative effects on sperm viability, motility, mitochondrial function, the percentage of apoptotic spermatozoa, and sperm DNA integrity; these toxic effects were countered by Etanercept, a TNF-α inhibitor, *in vitro* [44]. Similarly, the level of IL-6, another marker of inflammation, was found to be elevated in the seminal plasma of infertile males [45–47]. Some compounds have been shown to prevent D-gal-induced reproductive injury by inhibiting inflammation markers such as TNF-α and IL-6 [48]). The results of the present study provide evidence that GSLS might alleviate aging spermatogenic dysfunction by inhibiting inflammation.

Studies have shown that the activation of MAPKs signaling pathway is associated with testicular tissue injury such as depletion of germ cells and irregular small seminiferous tubule, as well as sex hormone secretion and sperm quantity and motility, the mechanism is related to MAPKs signal pathway involved in oxidative stress, inflammation and apoptosis [49–53]. In this study, we also observed activation of the MAPKs signaling pathway with vacuolar degeneration, germinal layer destruction, spermatogenic cell depletion, abnormality of sperm motility and changes of sex hormone levels. The above indicators were improved after treatment with GSLS.

In conclusion, we found that treatment with GSLS attenuated the negative effects of D-gal on reproductive hormones, sperm parameters and testis histopathology in male mice. At the same time, GSLS treatment inhibits the activation of MAPKs signaling pathways in testicular tissue. GSLS also elevated the activities of SOD and GSH-Px, decreased the contents of MDA and 8-OHdG, and reduced the levels of TNF-α, IL-1β and TNF-β1 in serum, liver and kidney compared to the D-gal-treated mice. The results suggest that GSLS have protective effects on reproductive function in aging male mice possibly by inhibiting the activation of MAPKs signal pathway in testis and reducing oxidative stress and inflammation. GSLS thus show promise for the construction of medicines to prevent aging-related reproductive dysfunction. However, the exact molecular targets of GSLS in the impairment of aging-related spermatogenesis remain obscure. Further research should be carried out to determine the molecular targets of GSLS.

## MATERIALS AND METHODS

### Chemicals and reagents

D-gal (≥99%) was purchased from Sigma Aldrich (St. Louis, MO, USA). Superoxide dismutase (SOD), glutathione peroxidase (GSH-Px), malondialdehyde (MDA), and 8-hydroxy deoxyguanosine (8-OHdG) commercial kits were purchased from Nanjing Jiancheng Institute of Biotechnology (Nanjing, China). Testosterone, cortisol, follicle-stimulating hormone (FSH), luteinizing hormone (LH), interleukin-4 (IL-4), interleukin-6 (IL-6), interleukin-1β (IL-1β), tumor necrosis factor-β1 (TNF-β1), and tumor necrosis factor-α (TNF-α) enzyme-linked immunosorbent assay (ELISA) kits were obtained from Nanjing SenBeiJia Biological Technology Co., Ltd (Nanjing, China). Anti-p-p38, anti-p-ERK1/2, anti-p-JNK, anti-p38, anti-ERK1/2 and anti-JNK antibodies were purchased from Bioss (Beijing, China). The primary antibody for GAPDH was from Biogot Technology Co., Ltd. (Nanjing, Jiangsu, China). Anti-rabbit secondary antibody was obtained from Cell Signaling Technology (Danvers, MA, USA). All other reagents used in the experiments were of analytical grade.

### Preparation of GSLS

GSLS were obtained from Acetar Bio-Tech, Inc. (Xi’an, China). The GSLS were prepared according to the method in Chinese Pharmacopoeia 2015. Dried ginseng stem-leaf powder was decocted twice with water, abstraction and separation for 2 h, 1.5 h, respectively. The two water decoctions were combined, then fractionated on a macroporous adsorption resin D l01 column with water and 60% ethanol, then concentrated and dried, finally the total ginsenoside extract from the stems and leaves was obtained. Total ginsenosides were quantificated using ultraviolet spectrophotometer as described in the Chinese Pharmacopoeia 2015. The content of GSLS was 30.24%.

### Animals treatment

The animal use protocol was approved by the Institutional Animal Care and Use Committee (IACUC) of Nanjing Medical University (Approval Number IACUC-1812019). Male ICR mice (18–25 g) were provided by the Animal Center of Nanjing Medical University (Nanjing, Jiangsu, China) and housed under specific pathogen-free conditions throughout the experimental period. The mice were permitted free access to food and water. The animals were kept at a temperature of 22° C ± 2° C with relative humidity of 50% ± 10% with 12-h light and dark periods.

After acclimatization for one week, the mice were randomly divided into four groups (*n* = 10 per group): the D-gal model group (MG; mice subcutaneously injected with D-gal at 300 mg/kg/day); normal group (NG; mice subcutaneously injected with an equal volume of saline instead of D-gal); low-dosage GSLS group (GS-L; mice subcutaneously injected with D-gal at 300 mg/kg/day and intragastrically administered with GSLS at 50 mg/kg/day); and high-dosage GSLS group (GS-H; mice subcutaneously injected with D-gal at 300 mg/kg/day and intragastrically administered with GSLS at 250 mg/kg/day). GSLS was prepared into 5- and 25-mg/mL solutions, which were respectively and administrated to the mice in the GS-L and GS-H groups by gavage. The intragastric volume of each mice was not more than 0.3 mL.

After six weeks of treatment, blood samples were collected in microcapillary tubes from each mouse’s retro-orbital plexus. Next, all animals were anesthetized and sacrificed. Serum was isolated by centrifugation at 3000 ×g for 10 min and stored at −80° C for subsequent analysis. The liver and kidney of the mice were quickly removed, washed with phosphate-buffered saline, and stored at −80° C for future analyses.

### Measurements of liver and kidney function

Liver and kidney function was assessed by estimating the levels of alanine-aminotransferase (ALT), aspartate-aminotransferase (AST), uric acid (UA), and serum creatinine (SCr) in the serum using an automatic biochemistry analyzer (HITACHI 7100, Hitachi Ltd, Tokyo, Japan).

### Measurements of oxidative stress and cytokines in serum and testes

Liver and kidney tissue were homogenised in 1/5 (w/v) 50 mM (pH 7.4) ice-cold phosphate buffered saline solution (PBS) containing a protease inhibitor cocktail (Sigma-Aldrich, St. Louis, MO) with 10 strokes at 1200 rev/min in a Potter homogeniser. Homogenates were directly centrifuged at 4000 rpm for 10 min to obtain the supernatant.

The levels of MDA, 8-OHdG, GSH-Px, and SOD in serum and testicular supernatant were measured using commercial kits according to the manufacturer’s instructions (Nanjing Jiancheng Institute of Biotechnology, Nanjing, China).

The concentrations of IL-4, IL-6, IL-1β, TNF-β1 and TNF-α in serum and testicular supernatant were tested using ELISA kits according to the manufacturer's instructions. At the end of all reactions, the absorbance of chromophore at 450 nm was determined using a microplate reader.

### Mouse sperm motility evaluation

At the end of the administration period, the animals were executed, and the sperm cells obtained from the cauda epididymis were collected into 1 mL of M199 culture medium containing 0.1% bovine serum albumin. Sperm motility was assessed after incubation for 30 min at 37° C. A sperm aliquot was placed on a pre-warmed 100-μm-deep counting chamber slide and analyzed using an IVOS sperm analyzer (HamiltonThorne Biosciences). The indicators used to monitor the quality of mouse sperm were recommended by the manufacturer and included average path velocity (VAP), straight line velocity (VSP); continuous line velocity (VCL), and amplitude of lateral head displacement (ALH).

### Measurements of sex hormone levels in serum

The levels of serum testosterone, cortisol, FSH and LH were tested using ELISA kits according to the manufacturer’s instructions (SenBeiJia Biological Technology Co., Ltd, Nanjing, China).

### Histopathology of testis

The mice were executed under anesthesia and testes were removed. The testes are washed with PBS, fixed in formalin, and inserted in paraffin. Slices of a few micrometres were cut, and then stained them with hematoxylin-eosin. The histopathological sections were observed under light microscope at a magnification of 400x.

### Western blotting

Testicular tissue was homogenized in radioimmunoprecipitation assay (RIPA) lysis buffe (Beyotime, Shanghai, China). Lysates from each sample were run on gels, electrotransferred onto polyvinylidene difluoride (Millipore, Billerica, MA, USA), immunoblotted with primary antibodies. Horseradish peroxidase (HRP)-conjugated goat anti-rabbit IgG was used as secondary antibody. Immunoreactive proteins were visualized by enhanced chemiluminescence (Cell Signaling Technology, USA).

### Statistical analysis

Data are expressed as the means ± SEM. Differences among groups were analyzed by one-way analysis of variance and the Student–Newman–Keul’s test using SPSS 22.0 software. Statistical significance was indicated by *p* < 0.05.

### Ethics approval and consent to participate

The animal use protocol was approved by the IACUC of Nanjing Medical University (Approval Number IACUC-1812019).
